# Size-dependent activity of silver nanoparticles on the morphological switch and biofilm formation of opportunistic pathogenic yeasts

**DOI:** 10.1186/s12866-020-01858-9

**Published:** 2020-06-22

**Authors:** Bettina Szerencsés, Nóra Igaz, Ákos Tóbiás, Zsombor Prucsi, Andrea Rónavári, Péter Bélteky, Dániel Madarász, Csaba Papp, Ildikó Makra, Csaba Vágvölgyi, Zoltán Kónya, Ilona Pfeiffer, Mónika Kiricsi

**Affiliations:** 1 Department of Microbiology, University of Szeged, Szeged, Hungary; 2 Department of Biochemistry and Molecular Biology, Faculty of Science and Informatics, University of Szeged, Közép fasor 52, Szeged, H-6726 Hungary; 3 Department of Applied and Environmental Chemistry, University of Szeged, Szeged, Hungary; 4 HAS-USZ Reaction Kinetics and Surface Chemistry Research Group, Szeged, Hungary

**Keywords:** Pathogenic yeasts, Silver nanoparticles, Morphological switch, Biofilm formation, Human keratinocytes, Toxicity

## Abstract

**Background:**

Dimorphism and biofilm formation are important virulence factors of some opportunistic human pathogenic yeasts. Such species commensally colonize skin or mucosal surfaces generally in yeast form, but under particular circumstances, convert into virulent hyphae and disseminate internal organs or cause mucocutaneous infections. The yeast-to-hypha shape-conversion promotes the development of a biofilm, a thick extracellular matrix with sessile cells within. The biofilm is capable to prevent the penetration of antifungal drugs, rendering the surviving biofilm-resident cells intrinsic sources of recurrent infections. The aim of this study was to evaluate the ability of silver nanoparticles (AgNPs) to attenuate the morphological switch and biofilm formation of several opportunistic pathogenic yeasts and to determine whether this feature depends on the nanoparticle size.

**Results:**

AgNPs in three different sizes were prepared by chemical reduction approach and characterized by transmission electron microscopy, ultraviolet–visible spectroscopy and dynamic light scattering. The antifungal activity was evaluated by the microdilution method, the inhibitory capacity on biofilm formation and the biofilm degradation ability of differently sized AgNPs was assessed by viability assay. The morphological state of opportunistic pathogenic yeast cells in monoculture and in co-culture with human keratinocytes in the presence of AgNPs was examined by flow cytometry and scanning electron microscopy. All the three AgNPs inhibited the growth of the examined opportunistic pathogenic yeasts, nevertheless, AgNPs with the smallest diameter exhibited the most prominent toxic activities. AgNPs attenuated the biofilm formation in a nanoparticle size-dependent manner; however, their biofilm destruction capacity was negligible. AgNPs with the smallest size exerted the most significant effect on suppressing the morphological change of pathogens in monoculture as well as in a co-culture with keratinocytes.

**Conclusions:**

Our results confirm that AgNPs are capable to hinder yeast-to-hypha morphological conversion and biofilm formation of opportunistic pathogens and this biological effect of AgNPs is size-dependent.

## Background

The incidence of human infections caused by yeasts or other fungi increased worldwide during the last decades, generating significant health problems [1]. Although the symptoms of fungal diseases are often mild and superficial (e.g. irritation, itching and swelling), on occasion, life-threatening invasive infections develop. The clinical manifestations of such infections are strongly related to host immunity and to the particular physiological condition of the patient [2, 3]. Despite antifungal therapy, invasive fungal diseases are associated with high mortality rate causing the death of about one and half million people in a year, mainly due to complications attributed to the infection [4]. Especially immune-compromised and severely ill patients are at high risk and exhibit increased susceptibility to systemic fungal infections [5]. The most common pathogens generating invasive infections belong to *Candida*, *Cryptococcus*, *Aspergillus* and *Pneumocystis* genera; however, several species from *Lodderomyces*, *Pichia, Rhodotorula* and *Trichosporon* genera have recently been implicated in the development of systemic infections [6–12].

Numerous factors influence the pathogenic capacity of the above mentioned species, such as secretion of specific enzymes, thermotolerance, toxin production, as well as morphologic transformation [2]. Indeed, several opportunistic pathogenic yeasts present dimorphism. Under certain conditions, these yeast cells experience a special morphological alteration and turn into elongated cells called hyphae. It has been shown that such a conversion is a fundamental feature of the pathophysiology and the virulence of these strains [13, 14]. As a matter of fact, opportunistic pathogens commensally colonize the skin or mucosal surfaces of the gastrointestinal and urogenital tracts mostly in yeast form, then yeasts switch to virulent hyphae which can disseminate internal organs or cause mucocutaneous infections [15].

Phenotypic switching requires a massive shift in the gene expression pattern to support the extensive remodeling of the cell wall and of the cytoskeleton, and to produce specific proteins to promote host adhesion and invasion. For example, hyphae are capable of releasing hydrolytic enzymes to help them penetrate into the host tissue more easily, but these filamentous cells can also assist other, non-dimorphic species upon host invasion [16]. Therefore, such a morphological switch represents an important virulence factor of the pathogens.

The yeast-to-hypha shape-conversion also promotes the development of a biofilm either on inert (medical devices, such as catheters, shunts and stents) or on biological surfaces, like skin or mucosa [17]. Biofilms are well-structured communities wherein cells are surrounded by a thick extracellular matrix [18]. Since this matrix prevents the penetration of drugs, sessile cells within the biofilms are usually protected from antifungal agents, thus the surviving biofilm-resident cells are intrinsic sources of recurrent infections [19].

The limited number and the moderate or low efficacy of antifungal drugs, the severe adverse effects related to their administration and the emergence of resistant strains are still main challenges in the treatment of fungal diseases, and stressed the need to develop new, highly effective drugs to combat pathogenic species. Silver nanoparticles (AgNPs) fulfill such requirements, since they exhibit outstanding biological activities, such as anti-cancer, antibacterial, antiviral and antifungal effects [20–25]. Exploiting these advantageous properties, the applications of AgNPs are widespread: as disinfecting materials, in cosmetic industry, wastewater treatment but also for a broad range of biomedical applications [20, 26]. As a consequence, there is a huge global demand to increase the production of nanosized silver, using conventional chemical methods or alternative, environmentally safe and sustainable, green synthetic approaches [27–31].

It was demonstrated on different human cell lines and even on plant and bacterial cells, that the biological effect of silver nanoparticles depends on the particle size [32–36]. Although the antifungal activity of AgNPs was proven on numerous species, the impact of nanoparticle size on the morphological switch of dimorphic yeasts has not been addressed yet. Since yeast-to-hypha shift is a significant virulence factor and is directly related to biofilm formation, it is fundamental to understand how the size of AgNPs could affect this feature. Therefore, in this present study we carried out a complex screening on selected dimorphic yeasts to determine the size-dependent effects of AgNPs on morphological change of these species. For this purpose, we synthesized AgNPs in three different sizes and treated dimorphic yeasts with the obtained nanoparticles. The morphology of the cells, the degree of biofilm development and the viability of the cells within the biofilm were analyzed after silver nanoparticle applications. To evaluate whether the effects of differently sized AgNPs on yeast-to-hypha switch manifest on biotic surface colonizing species as well, a co-culture system consisting of human keratinocyte cells and dimorphic yeasts was established and treated with AgNPs.

## Results

### Characterization of nanoparticles

Silver nanoparticles in three different sizes were prepared by chemical reduction in a size-controlled, seed-mediated growth approach and were subsequently characterized by transmission electron microscopy (TEM), ultraviolet–visible (UV-Vis) spectroscopy and dynamic light scattering (DLS).

**Fig. 1:**
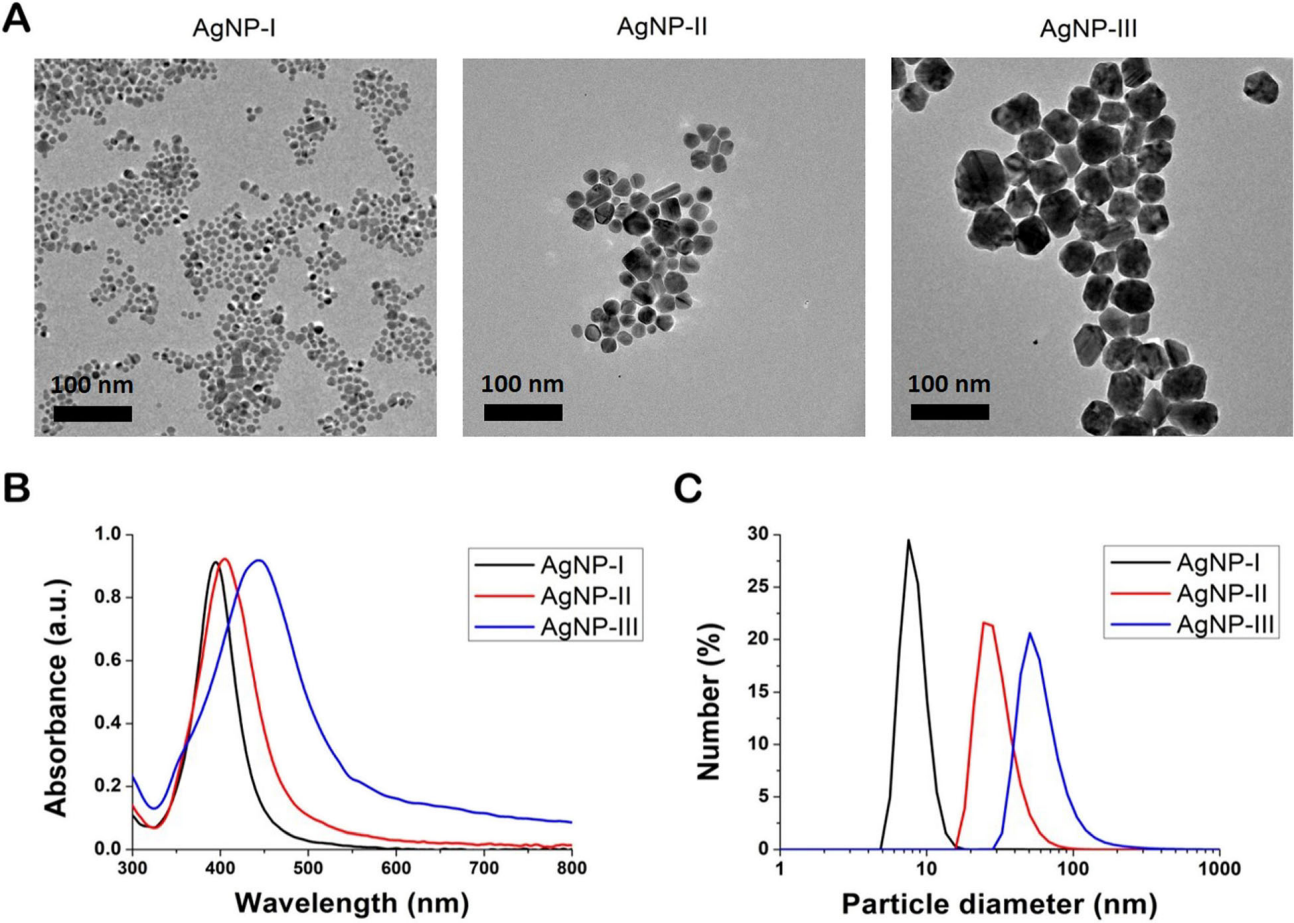
Physicochemical properties of citrate-stabilized silver nanoparticles of three different sizes. Transmission electron microscopic images (**a**) and UV-Vis spectra (**b**) of the synthetized, differently sized AgNPs. Size distribution of the nanoparticles determined by dynamic light scattering measurements (**c**)

TEM images indicated that all three nanoparticle samples contained quasi-spherical AgNPs (Fig. 1a). Based on image analysis only minor polydispersity was observed and the average size of the AgNPs proved to be 7.0 ± 1.6 nm for AgNP-I, 21.4 ± 4.6 nm for AgNP-II and 50.3 ± 7.9 nm for AgNP-III, respectively. UV-Vis spectra of AgNPs showed absorption peak maxima at 395 nm in case of AgNP-I, at 404 nm for AgNP-II and at 442 nm for AgNP-III, characteristic to surface plasmon resonance, which further supported the formation of metallic silver nanoparticles and confirmed the difference in average nanoparticle diameters between the three samples (Fig. 1b). According to DLS measurements (Fig. 1c), the average hydrodynamic particle size of AgNP-I was between 5 and 12 nm diameter, that of AgNP-II was between 15 and 50 nm, whereas the size of AgNP-III nanoparticles was around 35–90 nm, providing further evidence of the nanoparticle size difference in the obtained three silver colloid solutions.

### Antifungal activity of the differently sized AgNPs

The potential toxic effect of AgNPs with different sizes was tested against *Candida*, *Lodderomyces* and *Pichia* species (Table 1). We found that all three AgNP specimens inhibited the growth of the examined strains but in a different extent. AgNP-I proved to be the most effective one, while AgNP-III exerted the least toxic activity. *Pichia membranifaciens* CBS 191 was the most susceptible for the AgNPs as the lowest minimal inhibitory concentrations (MIC) were detected for this strain (Table 2). Unfortunately, in many cases the minimal inhibitory concentrations could not be established (Table 3) because of the low concentrations of the AgNP stock solutions.

**Table 1:** List of the tested strains

Species	Strain number
*Candida albicans*	SC 5314
*Candida dubliniensis*	CBS 7987
*Candida krusei*	CBS 573
*Candida parapsilosis*	CBS 604
*Candida tropicalis*	CBS 94
*Lodderomyces elongisporus*	CBS 1072
*Pichia membranifaciens*	CBS 191

*CBS* Centraalbureau voor Schimmelcultures, *SC* Squibb Institute for Medical Research, New Brunswick, New Jersey, USA

**Table 2:** Minimal inhibitory concentrations of the synthesized AgNPs against yeasts with pathogenic potential

Species	AgNP-I (μg/mL)	AgNP-II (μg/mL)	AgNP-III (μg/mL)
***Candida albicans*SC 5314**	**> 75**	**> 75**	**> 75**
***Candida krusei*CBS 573**	**> 75**	**> 75**	**> 75**
***Candida parapsilosis*CBS 604**	**37.5**	**> 75**	**> 75**
***Candida tropicalis*CBS 94**	**> 75**	**> 75**	**> 75**
***Candida dubliniensis*CBS 7987**	**> 75**	**> 75**	**> 75**
***Lodderomyces elongisporus*CBS 1072**	**37.5**	**18.75**	**37.5**
***Pichia membranifaciens*CBS 191**	**4.6875**	**18.75**	**37.5**

**Table 3:** The inhibition rate (%) in the presence of differently sized AgNPs applied in 75 μg/mL concentration

Species	AgNP-I	AgNP-II	AgNP-III
***Candida albicans*SC 5314**	**75**	**50**	**25**
***Candida krusei*CBS 573**	**90**	**90**	**10**
***Candida parapsilosis*CBS 604**	**100**	**90**	**65**
***Candida tropicalis*CBS 94**	**90**	**40**	**40**
***Candida dubliniensis*CBS 7987**	**60**	**20**	**0**
***Lodderomyces elongisporus*CBS 1072**	**100**	**100**	**100**
***Pichia membranifaciens*CBS 191**	**100**	**100**	**100**

### The effect of AgNPs on biofilm formation and destruction

In order to examine whether AgNPs are capable of modulating the propensity of dimorphic yeasts to build biofilms, secondly, to test whether the degree of such a modulatory action would depend on the nanoparticle diameter, we treated *Candida* and *Lodderomyces* cells with differently sized AgNPs in two concentrations and detected the viability of fungal cells within the biofilms by 2,3-bis-(2-methoxy-4-nitro-5-sulfophenyl)-2H-tetrazolium-5-carboxanilide (XTT) assay. The higher dose corresponded to 75 μg/mL in case of *C. albicans, C. dubliniensis, C. krusei, C. tropicalis*, 37.5 μg/mL for *C. parapsilosis* and 18.75 μg/mL for *Lodderomyces* sp., respectively. The lower dose was generally set as 18.75 μg/mL, except for *Lodderomyces elongisporus,* where it was set to 9.3 μg/mL.

**Fig. 2:**
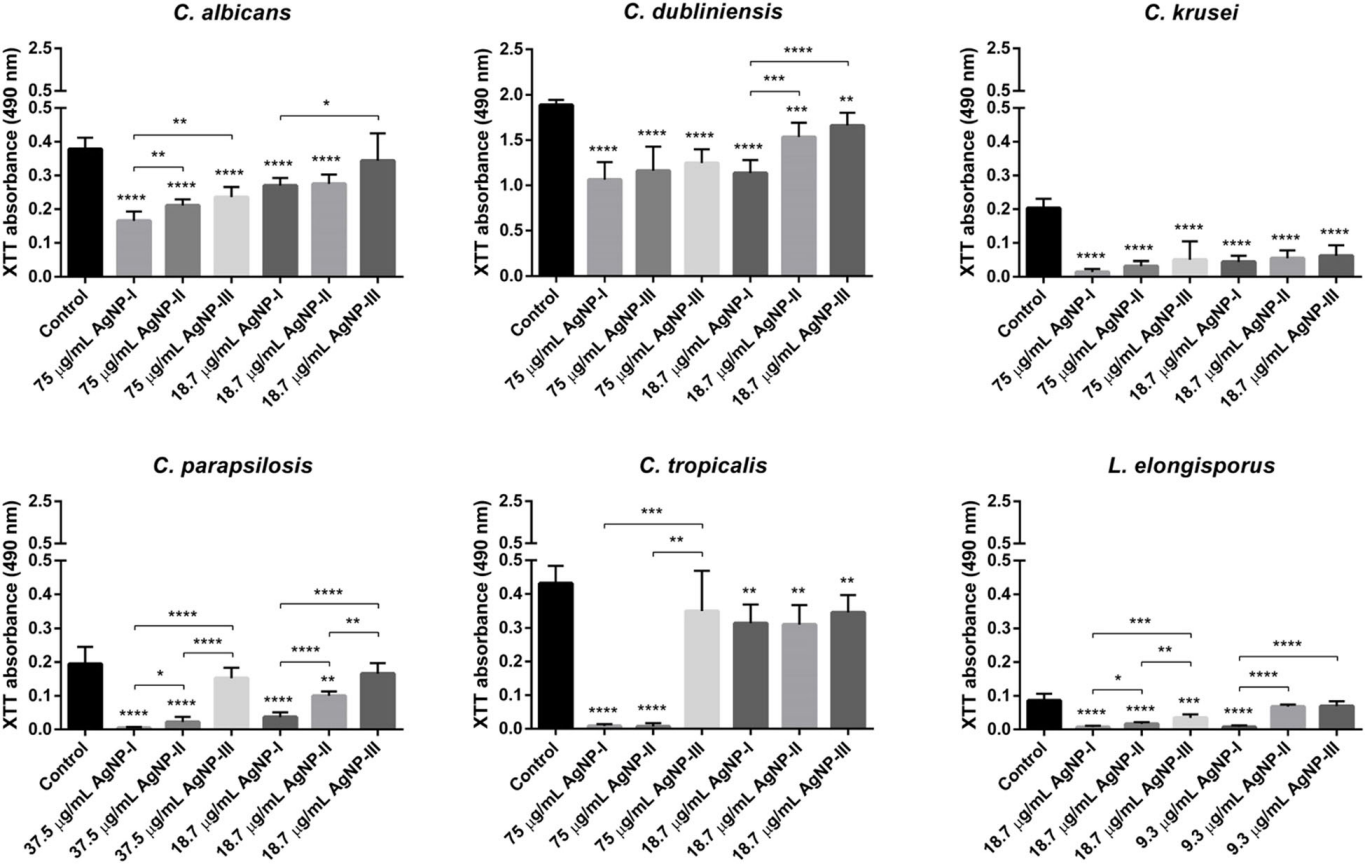
Inhibitory effect of differently sized AgNPs on biofilm formation of opportunistic pathogenic yeasts. Inhibitory effect of AgNPs on the formation of biofilms was evaluated by determining the viability of the various *Candida* and *Lodderomyces* cells in the biofilm using XTT assay after 72-h treatments with AgNP-I, AgNP-II or AgNP-III in the indicated concentrations. Suspensions without AgNPs were used as growth control. The values represent the mean ± standard deviation calculated from three independent experiments (*, *p* ≤ 0.05, **, *p* ≤ 0.01, ***, *p* ≤ 0.001, ****, *p* ≤ 0.0001, unpaired *t* test)

AgNPs exerted inhibitory effect on the biofilm formation of every examined *Candida* and *Lodderomyces* strain. In most cases the observed repression on biofilm development was dose- and size-dependent, and generally, nanoparticles with the smallest average diameter (AgNP-I) hampered the biofilm formation more efficiently than AgNP-II or AgNP-III solutions (Fig. 2). Moreover, treatments of the biofilms with AgNPs in higher concentrations could enhance their inhibitory effect on biofilm development (Fig. 2). *Pichia membranifaciens* CBS 191 cells did not adhere to the surface of the microplates, and did not develop a biofilm suitable for examination.

**Fig. 3:**
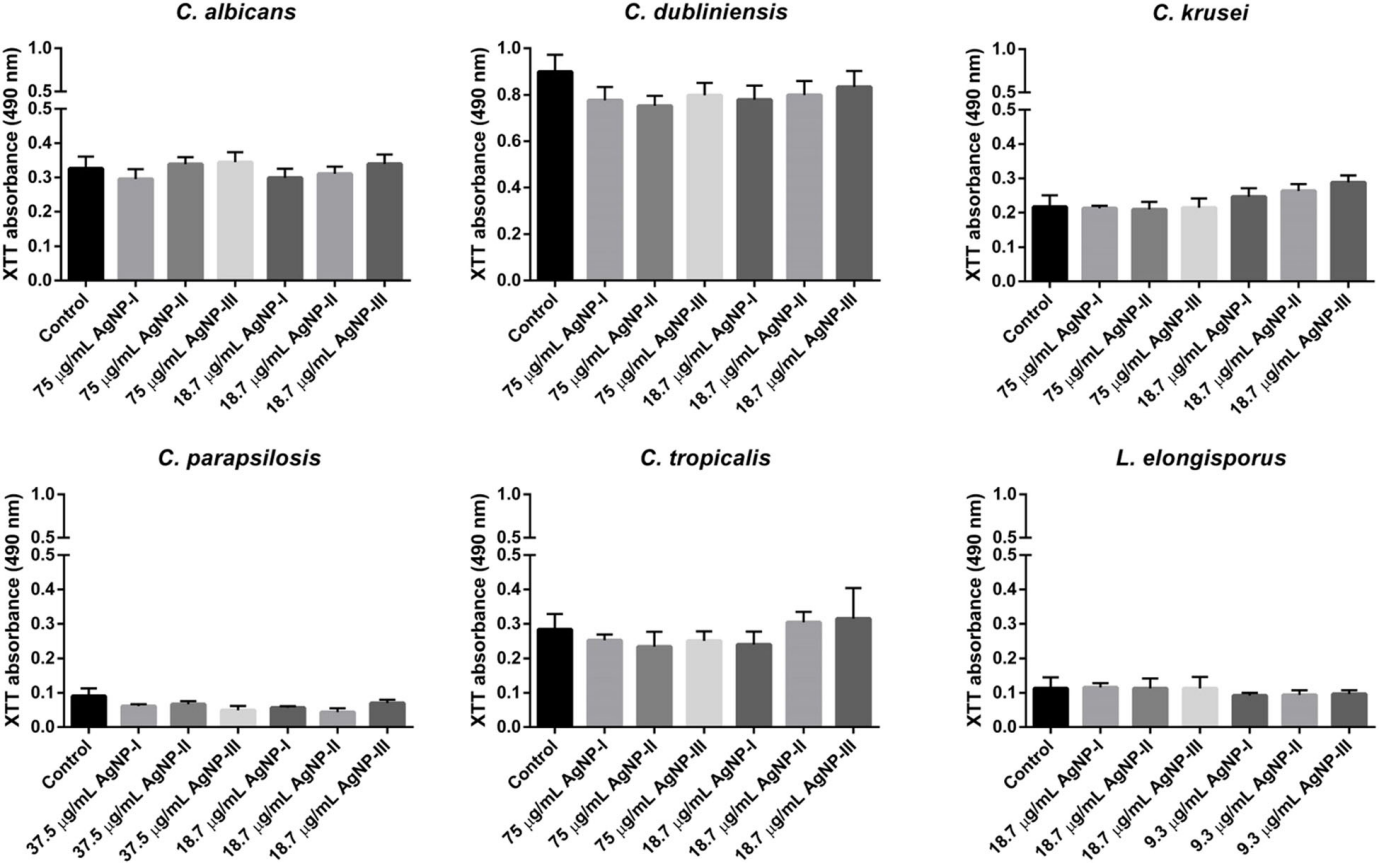
Effect of differently sized AgNPs on biofilm degradation. To determine the ability of AgNPs to destroy existing biofilms, three-day old matured biofilms of *Candida* and *Lodderomyces* strains were treated for 72 h with differently sized AgNPs in the indicated concentrations and the viability of the cells was assessed by XTT assay. The values represent the mean ± standard deviation calculated from three independent experiments

As we found that AgNPs inhibited biofilm formation quite efficiently, next we examined whether AgNPs were capable to destroy already existing fungal biofilms as well. The biofilm degradation ability of differently sized AgNPs was screened using matured biofilms of *Candida* and *Lodderomyces* species. Silver nanoparticles were applied on biofilms in two concentrations (the same high and low doses were used as upon biofilm formation experiments). Our results indicated that significant biofilm degradation could not be achieved using any of these citrate-stabilized AgNPs. Smaller nanoparticles applied in high concentration (AgNP-I, 75 μg/mL) were just as non-effective on the stable fungal biofilm as large AgNPs (Fig. 3).

### Morphology of AgNP-treated strains

**Fig. 4:**
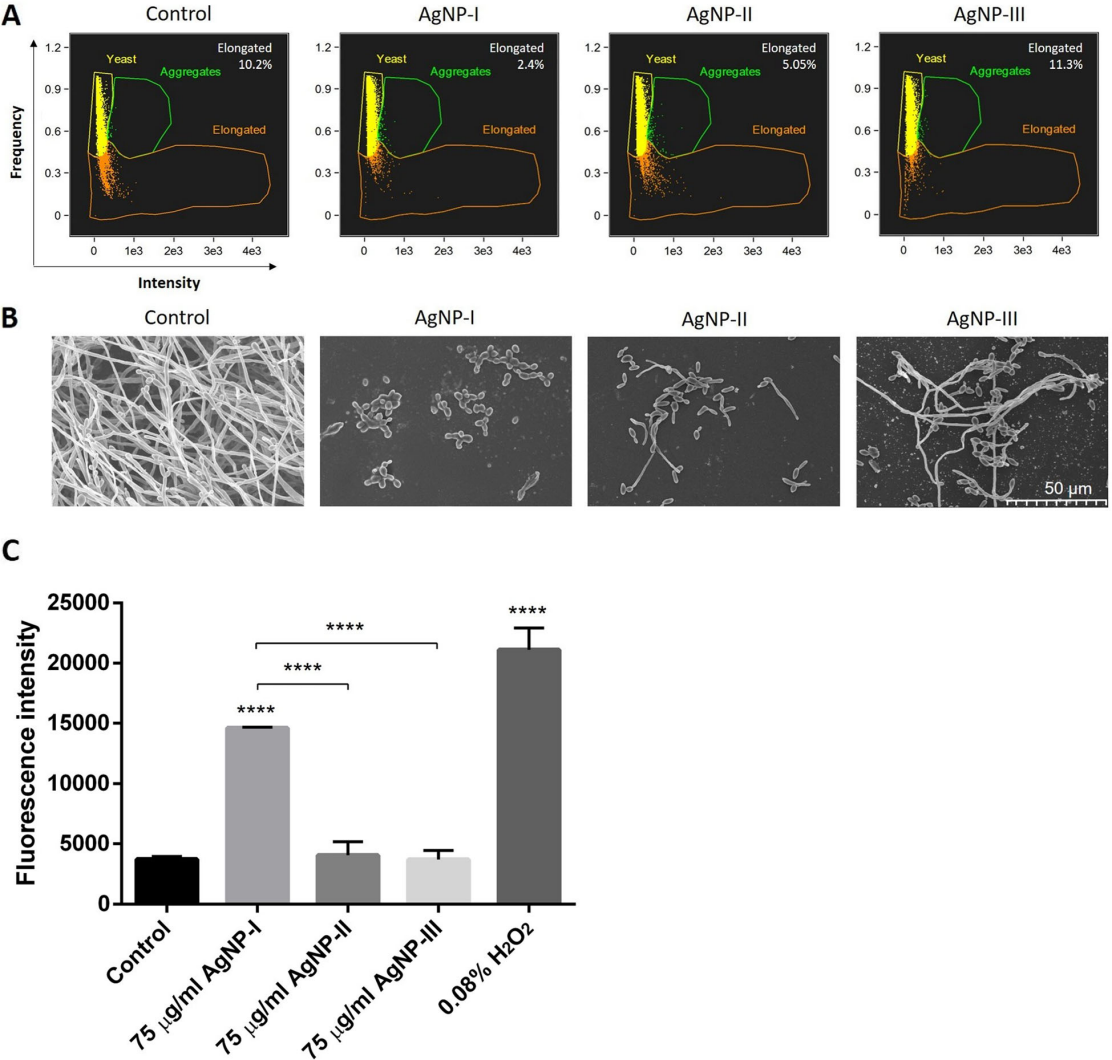
Morphology of control and AgNP-treated *C. albicans* cells. Cell morphology of untreated and AgNP-exposed *C. albicans* was detected by flow cytometry after 72-h incubation in the presence of differently sized AgNPs in 75 μg/mL concentration. The percentage of aggregated, yeast-shaped and elongated cells were quantified by IDEAS 6.2 software (Amnis-EMD Millipore, Burlington, MA, USA) and the percentage of elongated cells is indicated on the figures (**a**). Morphology of the surface-anchored cells after 72-h incubation in the presence of AgNPs was analyzed using scanning electron microscopic images (**b**). In order to assess the degree of reactive oxygen species formation, mean DCF fluorescence intensity was determined on DCFDA-stained, AgNP-treated *C. albicans* cells. Values represent the mean ± standard deviation calculated from three independent experiments (****, *P* < 0.0001, unpaired *t* test) (**c**)

Since the generation of elongated cells by yeast-to-hypha conversion is a prerequisite of biofilm formation by most dimorphic yeasts, the shape of *C. albicans* (Fig. 4) cells treated with differently sized AgNPs were analyzed first by flow cytometry, then with scanning electron microscopy (SEM). Using flow cytometry, the almost spherical yeasts can be discriminated from the elongated hypha in the supernatant of the sample, thus the differing morphological states of potentially pathogenic species can be quantified in suspension. By SEM on the other hand, the morphology of surface attached individual cells or cells within the biofilm can be visualized. The percentage of elongated *C. albicans* hyphae in the suspension of the untreated control was 10.2%, whereas in the supernatant of AgNP-I-treated samples only 2.4% of the cells were in hypha form. We quantified 5.05% elongated cells in the AgNP-II-, and 11.3% in the AgNP-III-treated sample supernatants by flow cytometry (Fig. 4a). These results indicate that small sized AgNPs (AgNP-I) were highly effective in suppressing the morphological conversion of dimorphic yeasts. On the other hand, the largest AgNPs (of approx. 50 nm diameter) had no effect on yeast-to-hypha transformation, as *C. albicans* cultures exposed to AgNP-III contained comparable amounts of elongated hypha forms in the supernatant as the untreated control. SEM images taken of AgNP-treated and untreated *C. albicans* cells (Fig. 4b) confirmed the morphological differences determined by flow cytometry. Mostly yeast forms were found in the AgNP-I-exposed samples, however, shorter or longer hyphae appeared in samples treated with AgNP-II and AgNP-III (representative images are shown in Fig. 4b). SEM images revealed massive biofilm formation in untreated *C. albicans* samples, where almost exclusively elongated hyphae were present. These results suggest that small-sized silver nanoparticles (AgNP-I) exhibit the highest potential to suppress yeast-to-hypha morphological switch of the opportunistic pathogenic yeast strains. Moreover, our data indicate that with increasing nanoparticle diameters the capacity of AgNPs to inhibit the morphological switch of dimorphic fungi decreases substantially.

Since the inhibition of morphological switch can be the consequence of oxidative stress induced by AgNP treatments, we investigated the reactive oxygen species (ROS) generating potential of differently sized AgNPs in *C. albicans* cultures. Cells were treated with AgNP-I, AgNP-II and AgNP-III, respectively, and were stained with 2′,7′–dichlorofluorescein diacetate (DCFDA). 2′,7′–dichlorofluorescein (DCF) fluorescence intensity values show that small-sized AgNPs (AgNP-I) induce significant ROS production compared to untreated control samples, however, medium-sized AgNPs are less potent and AgNP-III is a very weak inducer of ROS generation (Fig. 4c). Hence, the different degree of oxidative stress triggered by differently sized AgNPs can form the molecular basis for the morphology switch inhibiting potential of AgNPs in *C. albicans* cells.

### Efficiency of AgNPs to modulate the morphological switch of opportunistic pathogenic yeasts on biotic surfaces

The effects of differently sized AgNPs on yeast-to-hypha switch of opportunistic pathogenic yeasts were then determined in a co-culture system, consisting of human keratinocyte cells and dimorphic yeasts, generated with the aim to model fungal infection on biotic surfaces, like human skin.

First, the toxicity of the synthesized silver colloids was assessed on human HaCaT keratinocytes by 3-(4,5-dimethylthiazol-2-yl)-2,5 diphenyl tetrazolium bromide (MTT) viability assay. As expected, AgNPs decreased cell viability in size-, and concentration-dependent manner. The calculated half maximal inhibitory concentration (IC_50_) values indicated that the strongest toxicity on human keratinocytes was achieved upon small nanoparticle (AgNP-I) exposures (IC_50_ = 8.02 ± 1.7 μg/mL), while lower extent of cell death was induced by medium-sized AgNPs (AgNP-II, IC_50_ = 42.6 ± 18.3 μg/mL). The smallest decrease in viability was detected following AgNP-III nanoparticle treatments (IC_50_ = 91.3 ± 6.1 μg/mL).

**Fig. 5:**
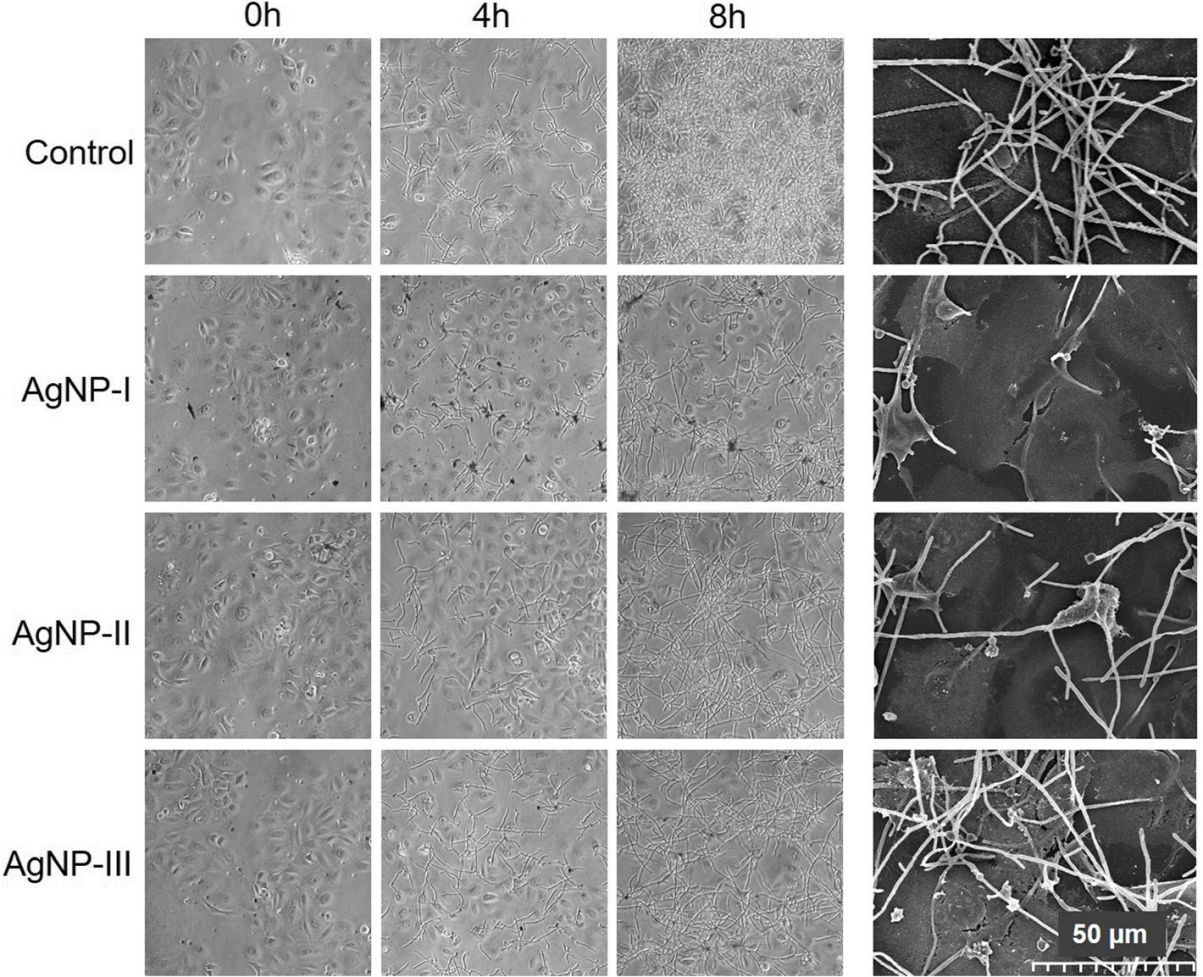
Size-dependent modulation of the morphological switch of opportunistic pathogenic yeasts co-cultured with keratinocytes by AgNPs. Morphology of *C. albicans* cells in co-culture with keratinocytes was monitored by light microscopy after 4 and 8 h, as well as by scanning electron microscopy after 8 h of AgNP-I, AgNP-II and AgNP-III treatments

Then, co-cultures of HaCaT human keratinocytes and *C. albicans* cells (in 1:5 human/yeast cell ratio) were established, and exposed to differently sized AgNPs in 75 μg/mL concentration for 8 h. During treatment hypha formation of *Candida* cells was followed microscopically. *C. albicans* cells were present in filamentous form in the untreated co-cultures, and after 8-h incubation a thick biofilm could be observed over and around keratinocytes (Fig. 5). AgNP treatments clearly reduced hypha formation of *C. albicans,* since less elongated cells were found in AgNP-exposed cultures after 4 and 8 h incubation, compared to the corresponding untreated control. Importantly, inhibition of the yeast-to-hypha switch in the co-culture proved to be nanoparticle size-dependent. We noticed that AgNP-I administration attenuated hypha formation as well as the biofilm generation of *C. albicans* with the highest degree on the surface of the keratinocyte layer. This inhibitory effect was smaller when AgNP-II was applied, and resulted to be the least pronounced upon AgNP-III administration (Fig. 5). SEM images taken from AgNP-treated *C. albicans* and HaCaT keratinocyte cells in the co-culture supported the above described morphological differences (Fig. 5). It is important to note that throughout the co-culture experiments, also upon AgNP treatments (8-h incubation of the co-cultures with AgNPs was monitored microscopically) keratinocyte morphology, the integrity of HaCaT cell layer as well as cell-cell contacts were not compromised. All these results indicate that the morphological switch of dimorphic, potentially pathogenic fungal cells can be attenuated by AgNPs not only on abiotic, but also on biotic surfaces such as skin, and smaller particle diameters are more advantageous to suppress the formation of virulent hyphae.

## Discussion

Morphological plasticity, such as the capability to undergo a yeast-to-hypha transformation, contributes significantly to the virulence of many opportunistic human pathogenic yeasts. In response to the changing environmental conditions within the host, planktonic yeast cells convert to filamentous hyphae, and the emerging elongated cells facilitate the pathogen penetration and potentiate host invasion [37]. Filament formation is also an indispensable factor for the generation of structured microbial communities called biofilm. Hyphae provide the structural integrity and the multilayered architecture for mature biofilms [38]. Biofilm-located cells express decreased sensitivity to antifungal compounds complicating the treatment of fungal diseases. Therefore, the inhibition of the morphological switch and thereby biofilm development could decrease the virulence of these pathogens and promotes the eradication of the infection.

The antiseptic application of silver goes back to ancient times, however, the emergence of various antibiotics confined its usage substantially [39]. In the last two decades, silver has been re-introduced to broad range medical applications in the nanoparticle formulation. AgNPs perform well against numerous Gram-positive and Gram-negative bacteria, several viruses and pathogenic fungi as well. The mechanism of action behind the anti-pathogenic activity of AgNPs has not been elucidated in every detail. Nevertheless, it is generally accepted that AgNPs exert their antimicrobial effects through microbial membrane damage, caused by the attachment of AgNPs on the cell surface, which leads to structural as well as functional alterations in the membrane (membrane destabilization, formation of pores and cytoplasm leakage). This action is complemented by the release of Ag^+^ ions from the nanoparticle surface, which provokes the generation of reactive oxygen species [39]. The latter will ultimately induce sub-cellular structure damage by inactivation and denaturation of essential microbial macromolecules (proteins, enzymes, and nucleotides) and leads to apoptotic cell death [40].

Although innovative AgNP synthesis methods emerge daily, and the as-prepared nanomaterials are now routinely tested for cytotoxic, antibacterial or antifungal activities [41–43], the scientific literature is scarce in reports describing the effects of AgNPs on yeast cell ultrastructure and biofilm development. In fact, only a few authors examined the AgNP-induced morphological changes of *Candida albicans*. They demonstrated that silver nanoparticles hamper *C. albicans* biofilm formation in vitro via inhibition of filament formation and by destruction and of the cell wall integrity. Lara and co-workers applied extremely small, in average 1 nm sized AgNPs on *C. albicans* cells [38], whereas Muthamil et al. used relatively big (40–55 nm) AgNPs [44], and finally, Jalal et al. utilized polydispersed biosynthesized AgNPs [45]. However, none of these studies investigated the size-dependent effect of the AgNPs on this species, nor tested the actions of AgNPs on dimorphic yeasts of other genus.

Therefore, in the present work we studied the size-dependent action of silver nanoparticles on the morphological transition and subsequent biofilm formation of different dimorphic yeast species. To achieve this aim citrate-coated AgNPs in three different sizes (AgNP-I: ~ 7.0 nm; AgNP-II: ~ 21 nm; AgNP-III: ~ 50 nm) were successfully prepared by one of the most widely utilized chemical reduction approaches [46]. The sizes of the different AgNP samples were verified by TEM and DLS measurements. UV-Vis spectra of the prepared colloids also supported the synthesis of AgNPs based on previously reported spectral characteristics of particles with similar morphologies [47]. The antifungal activity test revealed that silver nanoparticles with the smallest size (AgNP-I) were the most effective against the examined opportunistic pathogenic species. *C. parapsilosis* and rare pathogens such as *L. elongisporus* and *P. membranifaciens* proved to be the most sensitive to AgNP exposures [48].

Importantly, biofilm development of the *Candida* species and *L. elongisporus* was substantially inhibited in the presence of the nanoparticles, and in this respect again AgNP-I proved to be the most potent, as treatments with small nanoparticles led to the most significant decline in the viability of biofilm-located cells. The strong capacity of small AgNPs to inhibit biofilm formation correlated well with the attenuated hypha formation of AgNP-I-exposed dimorphic yeasts, demonstrated by FACS and SEM data. Most probably the formation of reactive radicals and the consequent oxidative stress induced by small AgNPs lay behind this outstanding potential of small-sized nanoparticles to hamper morphological switch in dimorphic yeasts.

The observed inhibitory feature of AgNPs on yeast-to-hypha morphological conversion holds great potential in nanomedicine, if this ability of AgNPs manifests on biological surfaces. Therefore, we tested the capability of AgNPs to suppress the morphological transformation of *C. albicans* in a co-culture system with human keratinocytes. As before, considerably decreased hypha formation was detected in the presence of AgNP-I compared to untreated control cultures. These results suggest that the capability of AgNPs, especially those with small diameters, to modulate the morphological transformation and thereby the virulence of dimorphic pathogenic yeasts is maintained on biotic surfaces, like skin and mucosa, which feature can be exploited further in biomedical applications. On the other hand, it is important to note, that in contrast to the findings by Lara et al. [38], in our study AgNPs could not disintegrate already developed, matured biofilms, since we found that the biofilm destruction ability of the differently sized, citrate-coated AgNPs was negligible. Our findings imply the prophylactic application of citrate-coated AgNPs in order to take advantage of their antifungal capacities against dimorphic yeasts.

Normally, a sequence of tightly regulated events leads to successful biofilm generation. Yeast cells colonize and adhere to soft or hard surfaces mediated by specific adhesion factors. In case of *C. albicans*, key regulators, like Bcr1, as well as its downstream targets such as cell wall proteins Als1, Als3 have been implicated as required elements for the early steps of the process [49]. Cell-cell communication, especially quorum sensing, is also crucial at this stage to prevent overpopulation and competition for nutrients, and for the induction of hypha-formation-associated genes. After initial adherence and focal attachment of individual cells, a basal layer is formed, which is then subjected to cell proliferation, early-stage filamentation, followed by the production of exopolymeric substance by sessile cell communities in the multilayered network regulated by factors like Rlm1 and Zap1 in *C. albicans* [49]. Exposure of dimorphic fungi to AgNPs and the produced reactive radicals might affect most of these events, nevertheless, the precise mechanisms at the molecular level behind the inhibitory action of AgNPs on the morphological switch and biofilm formation of these opportunistic pathogens is yet to be demonstrated. In fact, we have already started to establish a set of mutant *Candida* strains for testing which molecular event related to morphological conversion is perturbed by AgNP exposures or by the triggered oxidative stress.

Our results indicate that virulence factors of opportunistic pathogens, like morphological switch and biofilm formation can be efficiently modulated by the utilization of AgNPs. However, it has to be stressed that in order to achieve the desired effects, nanomaterials have to be designed properly. We found that size indeed matters when AgNPs are intended to act on dimorphic pathogenic yeasts. The performance of larger nanoparticles (AgNP-III) in impairing yeast-to-hypha conversion and biofilm generation was considerably worse than medium (AgNP-II) and small-sized (AgNP-I) counterparts, which phenomenon might be explained by their different abilities in ROS production. Since AgNP size seems to have a defining role in determining and fine-tuning the biological activity of nanoparticles, hence, more careful considerations are required upon nanomaterial design and production and thorough screening regimens of the obtained nanomaterials are recommended to estimate their efficiencies.

## Conclusions

Our data confirm that citrate-coated AgNPs are able to inhibit the morphological switch and biofilm formation of dimorphic yeasts, however, they are not effective against fully developed biofilms. AgNPs with the smallest size performed especially well in all experiments further supporting the fact that particle size has significant impact on the biological activity of these nanomaterials. Our results might influence potential strategies and opportunities for the clinical translation of AgNP application in the field of medical mycology.

## Methods

### Synthesis of silver nanoparticles

All chemicals for the nanoparticle synthesis were obtained from Merck (Darmstadt, Germany).

Citrate-stabilized silver nanoparticles in three different sizes were prepared by chemical reduction using sodium borohydride, according to Wan et al. with slight adjustments [46]. Twenty mL of 1% w/v citrate solution and 75 mL of water were added in a beaker and the mixture was heated to 70 °C. Then, 2 mL of 1% w/v AgNO_3_ solution were introduced to the mixture, followed by drop-wise addition of 2 mL of 0.1% w/v freshly prepared sodium borohydride solution. The reaction solution was kept at 70 °C under vigorous stirring for 1 h and was then cooled to room temperature. The resulting AgNPs, denoted as AgNP-I, were used as starter seeds for AgNP-II.

To obtain larger AgNPs in a size-controlled manner, a seed-mediated growth approach was employed. For the synthesis of AgNP-II with an average diameter of 20 nm, 2 mL of 10% w/v citrate solution was mixed with 75 mL of water and brought to 80 °C. Next, 10 mL of starter seed solution (AgNP-I) were added, followed by the supplementation of 2 mL of 1% w/v AgNO_3_ solution while vigorous mechanical stirring for 2 h, then the resulting suspension was cooled to room temperature.

For the production of AgNP-III with an average size of 50 nm, the 20 nm AgNPs (AgNP-II), obtained in the previous synthesis procedure, were used as seeds, and the above described growth procedure steps were repeated. The final colloid samples were stored at 4 °C in all cases.

After synthesis, the nominal concentrations of the three AgNP samples were about 128 μg/mL, 157 μg/mL and 160 μg/mL, respectively. For an easier handling throughout the experiments, we decided to set the concentration of all three samples to 150 μg/mL. Concentrating AgNP-I was achieved by placing the sample in a drying oven on 40 °C overnight, then measuring its volume and adding distilled water if necessary, to get a final volume of about 85 mL. The latter two samples after similar procedures were diluted to 93 and 95 mL final volume respectively, thus the standard 150 μg/mL concentration for all three samples was achieved. The calculations (see supplementary material) show how the nominal concentrations could be assessed for each sample and the V_150_ values highlight the final volume of the standardized AgNP samples that were used for the subsequent in vitro experiments.

### Characterization of nanoparticles

The morphological characteristics of AgNPs were examined by transmission electron microscopy applying a FEI Tecnai G^2^ 20× microscope (FEI Corporate Headquarters, Hillsboro, OR, USA) at an acceleration voltage of 200 kV. The optical properties of nanoparticles were assessed by spectral analysis, where the absorbance spectra of nanoparticles were recorded using an Ocean Optics 355 DH-2000-BAL UV–Vis spectrophotometer (Halma PRC, Largo, FL, USA) within the 300–800 nm range. The size distribution of the nanoparticle samples was examined by DLS analysis using a Zetasizer Nano ZS Instrument (Malvern, Worcestershire, UK).

### Antifungal activity

The antifungal susceptibility test was carried out using the three differently sized silver nanoparticles (AgNP-I, AgNP-II and AgNP-III) on each potentially pathogenic yeast species listed in Table 1. The minimal inhibitory concentration (MIC) was determined with the microdilution method in 96-well microplates. To 50 μL of standardized cell suspension (5 × 10^4^ cell/mL in Dulbecco’s Modified Eagle Medium (DMEM) supplemented with 1% fetal bovine serum (FBS) 50 μL silver nanoparticle solution was added in serially two-fold-diluted concentrations (in the range of 75 and 4.69 μg/mL in the above described medium) and incubated for 48 h at 30 °C (20 °C in the case of *Pichia membranifaciens*). All *Candida* and *Lodderomyces* species grow well at 30 °C, however, the growth of *C. parapsilosis* and *Lodderomyces elongisporus* is somewhat hampered at 37 °C. Thus, for this experiment 30 °C incubation temperature was applied to establish the MIC values in vigorously growing cultures. The control samples contained 50 μL cell suspension and 50 μL medium without nanoparticles. The optical density of the cultures was detected at 620 nm in SPECTROstar Nano plate reader (BMG LabTech, Offenburg, Germany). The experiments were carried out at least three times always in triplicates.

### Effect of AgNPs on biofilm formation

The biofilm formation and the viability of the cells in the biofilm were tested after AgNP treatments. Briefly, suspensions from each strain in 5 × 10^4^ cell/mL density were prepared in DMEM medium supplemented with 1% FBS. Then, 50 μL of the cell suspension were transferred into wells of a microtiter plate and 50 μl of either AgNP-I, AgNP-II or AgNP-III were added in 75 (high dose) or in 18.7 μg/mL (lower dose) concentrations, respectively. In some cases where preliminary experimental results indicated high susceptibility, the AgNP concentrations corresponding to high dose and/or low dose were set differently. Therefore, 37.5 and 18.7 μg/mL were applied for *C. parapsilosis* and 18.7 and 9.3 μg/mL concentrations for *L. elongisporus*. Suspensions without AgNPs were used as growth control, while the medium without cells and without AgNPs was used as negative control. Plates were incubated for 72 h at 37 °C in 5% CO_2_ level, then the biofilms were washed twice with phosphate buffered saline (PBS) to remove slightly attached cells and 2,3-bis-(2-methoxy-4-nitro-5-sulfophenyl)-2H-tetrazolium-5-carboxanilide (XTT) reduction assay was used to detect the viability of the biofilm-associated cells. XTT was prepared at 0.5 mg/mL concentration in PBS supplemented with 1 μM menadion. After adding 100 μL XTT solution to each well, the plates were incubated for 2 h at 37 °C in dark. Thereafter, 80 μL of each supernatant were transferred to new 96-well plates and the absorbance of each solution was measured at 490 nm using SPECTROstar Nano plate reader (BMG LabTech, Offenburg, Germany). The experiments were carried out three times always in 8 biological replicates.

### Determination of biofilm degradation ability

The biofilm formation was initiated by inoculating 100–100 μL cell suspensions (prepared in DMEM medium supplemented with 1% FBS in 5 × 10^4^ cell/mL density) from each strain into the wells of flat-bottomed 96-well microtiter plates. Plates were incubated for 72 h at 37 °C in an atmosphere containing 5% CO_2_. After biofilm formation, the medium was removed and silver nanoparticle solutions (diluted in DMEM medium) were added to the biofilms in 75 or 18.7 μg/mL final concentrations. For *C. parapsilosis*, high dose corresponded to 37.5 μg/mL while low dose was 18.7 μg/mL concentration and in case of *L. elongisporus* 18.7 and 9.3 μg/mL concentrations were set as high and low dose. Then plates were further incubated for 72 h at 37 °C. A series of only DMEM-treated biofilms and biofilm-free wells containing DMEM medium served as positive and negative controls. Viability of the cells was detected by XTT reduction assay, as described above. All the assays were carried out three times in 8 replicates.

### Morphological characterization of fungal cells

*C. albicans* was selected for morphological analysis of cells treated with differently sized AgNPs. *C. albicans* cells were inoculated into DMEM medium supplemented with 1% FBS in 5 × 10^4^ cell/mL concentration and were cultured for 24 h at 37 °C in the absence, as well as in the presence of either AgNP-I, AgNP-II or AgNP-III particles in 75 and 18.75 μg/mL final concentrations. The proportion of yeast cells and hyphae was determined from the sample supernatant by flow cytometry (FlowSight®, Amnis-EMD Millipore, Burlington, MA, USA). Cells cultured without AgNPs were used as control.

### Detection of reactive oxygen species

ROS production upon AgNP treatments was detected by DCFDA staining. *C. albicans* cells were inoculated into DMEM medium supplemented with 1% FBS in 5 × 10^4^ cell/mL concentration and were cultured for 24 h at 37 °C in the absence, as well as in the presence of either AgNP-I, AgNP-II or AgNP-III particles of 75 μg/mL final concentrations. Cells cultured with H_2_O_2_ in 0.08% concentration were used as positive control. After the treatments all cultures were incubated with DMEM containing 10 μM DCFDA (Sigma-Aldrich) in the dark for 1 h. The total fluorescence intensity of the samples was measured by flow cytometry (FlowSight®, Amnis-EMD Millipore, Burlington, MA, USA). Measurements were repeated three times using 3 independent biological replicates.

### Keratinocyte cell culture

HaCaT immortalized human keratinocyte cells were purchased from ATCC (Manassas, VA, USA) and maintained in DMEM medium containing 4.5 g/L glucose (Sigma-Aldrich, Saint Louis, MO, USA), supplemented with 10% FBS, 2 mM L-glutamine, 0.01% streptomycin and 0.005% penicillin (Sigma-Aldrich, Saint Louis, MO, USA). Cells were cultured in a 37 °C incubator at 5% CO_2_ in 95% humidity.

### Measurement of keratinocyte viability

HaCaT cell viability was measured after AgNP-I, AgNP-II and AgNP-III treatments. For this HaCaT cells were seeded into 96-well plates in 10,000 cells/well density, and treated on the following day with silver nanoparticles of three different sizes in increasing concentrations. After 24-h treatments, HaCaT cells were washed with PBS and incubated for an hour at 37 °C with 0.5 mg/mL MTT reagent (SERVA, Heidelberg, Germany) diluted in culture medium. Formazan crystals were solubilized in DMSO (Sigma-Aldrich, Saint Louis, MO, USA) and absorption was measured at 570 nm using a Synergy HTX plate reader (BioTek-Hungary, Budapest, Hungary). Experiments were performed at least three times using four independent biological replicates. IC_50_ values were obtained based on the results of MTT assays.

### Co-culture system

HaCaT immortalized human keratinocyte cells were used in a co-culture system together with *C. albicans* cells in order to model fungal infection of human skin. To investigate the hyphae formation of yeast cells within co-cultures upon AgNP treatments, HaCaT cells were seeded into 6-well plates and left to grow until they reached confluence to provide a continuous, consistent biosurface. Then, when this biofilm layer was completely established, 5 × 10^5^*C. albicans* cells were added to the wells, and the co-cultures were exposed to 75 μg/mL AgNPs. The cultures were continuously monitored under the microscope, throughout the investigation period (maximum 8 h). Photographs were taken after 0; 4; and 8-h treatments using Nikon Eclipse TS100 inverted light microscope (Nikon, Minato, Japan).

### Scanning electron microscopy

A.: The ultrastructure of the AgNP-treated and non-treated *C. albicans* biofilms formed on sterile glass coverslips (Sigma-Aldrich, Saint Louis, MO, USA) was examined by scanning electron microscopy. Biofilm formation was carried out as described above in the related Methods section. Cell suspensions were treated with either AgNP-I, AgNP-II or AgNP-III in 75 μg/mL concentration for 72 h at 37 °C, then cells were washed with PBS and fixed in 2.5% glutaraldehyde at 4 °C overnight.
B.: SEM images were taken of untreated as well as of AgNP-treated HaCaT - *C. albicans* co-cultures. For this, HaCaT cells were seeded onto plastic coverslips (Sarstedt, Nümbrecht, Germany) placed in 6-well plates. On the following day 5 × 10^5^*C. albicans* yeast cells were added to each well and the co-cultures were treated with differently sized AgNPs in 75 μg/mL concentration. After 8 h of treatment, cells were washed with PBS and fixed in 2.5% glutaraldehyde at 4 °C overnight.

All samples (prepared as described in part **A** or **B**) were dehydrated by increasing percentage of ethanol diluted in water (50, 70, 80, 90, 95, 98, 100%, for 15 min each), followed by a series of tert-butanol:ethanol mixture (1:2, 1:1, 2:1 volume ratio) at room temperature. Then the cells were incubated with absolute tert-butanol overnight at 4 °C and were finally lyophilized. The coverslips were mounted on specimen stubs with electrically conductive adhesive tape and the samples were covered with a thin metal palladium-gold layer. SEM imaging was performed by a Hitachi S4700 electron microscope (AuroScience, Budapest, Hungary) using 10 kV accelerating voltage and 10 μA emission current.

## Supplementary information

**Additional file 1.**

## Data Availability

All original data of this manuscript are available from the corresponding author on reasonable request.

## Abbreviations

AgNPs: Silver nanoparticles
CBS: Centraalbureau voor Schimmelcultures
DCF: 2′,7′–dichlorofluorescein
DCFDA: 2′,7′–dichlorofluorescein diacetate
DLS: Dynamic light scattering
DMEM: Dulbecco’s Modified Eagle Medium
FBS: Fetal bovine serum
IC50: Half maximal inhibitory concentration
MIC: Minimal inhibitory concentration
MTT: 3-(4,5-dimethylthiazol-2-yl)-2,5 diphenyl tetrazolium bromide
PBS: Phosphate buffered saline
ROS: Reactive oxygen species
SC: Squibb Institute for Medical Research
SEM: Scanning electron microscopy
TEM: Transmission electron microscopy
UV-Vis: Ultraviolet–visible
XTT: 2,3-bis-(2-methoxy-4-nitro-5-sulfophenyl)-2H-tetrazolium-5-carboxanilide
