# Supplementary material for: Size-dependent activity of silver nanoparticles on the morphological switch and biofilm formation of opportunistic pathogenic yeasts
Source: BMC Microbiol. 2020 Jun 22;20:176. doi: 10.1186/s12866-020-01858-9 (PMC7309973; doi:10.1186/s12866-020-01858-9)
Supplement: Supplementary file 1 — Additional file 1. [file 12866_2020_1858_MOESM1_ESM.docx]

**Supplementary material**

**Size-dependent activity of silver nanoparticles on the morphological switch and biofilm formation of opportunistic pathogenic yeasts**

Bettina Szerencsés^a§^, Nóra Igaz^b§^, Ákos Tóbiás^b^, Zsombor Prucsi^b^, Andrea Rónavári^c^, Péter Bélteky^c^, Dániel Madarász^c^, Csaba Papp^a^, Ildikó Makra^b^, Csaba Vágvölgyi^a^, Zoltán Kónya^c,d^, Ilona Pfeiffer^a#^, Mónika Kiricsi^b#^

*^a^Department of Microbiology, University of Szeged, Szeged, Hungary;*

*^b^Department of Biochemistry and Molecular Biology, University of Szeged, Szeged, Hungary;*

*^c^Department of Applied and Environmental Chemistry, University of Szeged, Szeged, Hungary;*

*^d^HAS-USZ Reaction Kinetics and Surface Chemistry Research Group, Szeged, Hungary.*

^§^ These authors contributed equally

**Corresponding authors (**^#^**):**

Mónika Kiricsi, PhD, Department of Biochemistry and Molecular Biology, Faculty of Science and Informatics, University of Szeged, Közép fasor 52. H-6726 Szeged, Hungary, Phone number: +36 (62) 544887, Fax: +36 (62) 544887, E-mail: [kiricsim@gmail](mailto:kiricsim@gmail).com

Ilona Pfeiffer, PhD, Department of Microbiology, Faculty of Science and Informatics, University of Szeged, Közép fasor 52. H-6726 Szeged, Hungary, Phone number: +36 (62) 544517, E-mail: [pfeiffer@bio.u-szeged.hu](mailto:kiricsim@bio.u-szeged.hu)

**Setting the unified concentration for the prepared AgNP dispersions**

After synthesis, the nominal concentrations of the three AgNP samples were about 128 ppm (µg/mL) for AgNP-I, 157 ppm (µg/mL) and 160 ppm (µg/mL), respectively for AgNP-II and AgNP-III. For an easier handling throughout the experiments, we decided to set the concentration of all three samples to 150 ppm (µg/mL). Concentrating AgNP-I was achieved by placing the sample in a drying oven on 40 °C overnight, then measuring its volume and adding distilled water if necessary, to get a final volume of about 85 mL. The latter two samples after similar procedures were diluted to 93 and 95 mL final volume respectively, thus the standard 150 µg/mL concentration for all three samples was achieved.

The calculations below show how the nominal concentrations could be assessed for each synthesized AgNP colloid sample and the V_150ppm_ values highlight the final volume of the standardized AgNP samples which were set for unification of concentration of the nanoparticle dispersions to150 ppm (µg/mL).

AgNP-I

$$V_{{total}_{(AgNP-I)}}=99 mL$$

$$m_{{AgNO}_{3} solution}=0,01\frac{g}{mL}*2 mL=0,02 g$$

$$m_{Ag solution}= \frac{m_{{AgNO}_{3} solution}}{M_{{AgNO}_{3}}}*M_{Ag}\approx0,0127 g=12700 \mu g$$

$$m_{{Ag}_{(AgNP-I)}}=m_{Ag solution}=12700 \mu g$$

$$c_{{nominal}_{(AgNP-I)}}= \frac{m_{{Ag}_{(AgNP-I)}}}{V_{{total}_{(AgNP-I)}}}\approx128 ppm$$

$$V_{{150ppm}_{(AgNP-I)}}= \frac{m_{{Ag}_{(AgNP-I)}}}{150 ppm}=84,66\dot{6} mL \approx85 mL$$

AgNP-II

$$V_{{total}_{\left( AgNP-II \right)}}=89 mL$$

$$m_{{Ag}_{\left( 10 mL AgNP-I \right)}}=10 mL*c_{{nominal}_{\left( AgNP-I \right)}}=1280 \mu g$$

$$m_{{Ag}_{(AgNP-II)}}= m_{Ag solution}+ m_{{Ag}_{\left( 10 mL AgNP-I \right)}}=13890 \mu g$$

$$c_{{nominal}_{\left( AgNP-II \right)}}= \frac{m_{{Ag}_{(AgNP-II)}}}{V_{{total}_{\left( AgNP-II \right)}}}\approx157 ppm$$

$$V_{{150ppm}_{(AgNP-II)}}= \frac{m_{{Ag}_{(AgNP-II)}}}{150 ppm}=92,6 mL \approx93 mL$$

AgNP-III

$$V_{{total}_{\left( AgNP-III \right)}}=89 mL$$

$$m_{{Ag}_{\left( 10 mL AgNP-II \right)}}=10 mL*c_{{nominal}_{\left( AgNP-II \right)}}=1570 \mu g$$

$$m_{{Ag}_{(AgNP-III)}}= m_{Ag solution}+ m_{{Ag}_{\left( 10 mL AgNP-II \right)}}=14270 \mu g$$

$$c_{{nominal}_{\left( AgNP-III \right)}}= \frac{m_{{Ag}_{(AgNP-III)}}}{V_{{total}_{\left( AgNP-III \right)}}}\approx160 ppm$$

$$V_{{150ppm}_{(AgNP-III)}}= \frac{m_{{Ag}_{(AgNP-III)}}}{150 ppm}=95,13 mL \approx95 mL$$
